# Improving prediction and assessment of global fires using multilayer neural networks

**DOI:** 10.1038/s41598-021-81233-4

**Published:** 2021-02-08

**Authors:** Jaideep Joshi, Raman Sukumar

**Affiliations:** 1 Centre for Ecological Sciences, Indian Institute of Science, Bangalore, 560012 India; 2 Divecha Centre for Climate Change, Indian Institute of Science, Bangalore, 560012 India

**Keywords:** Fire ecology, Climate-change ecology

## Abstract

Fires determine vegetation patterns, impact human societies, and are a part of complex feedbacks into the global climate system. Empirical and process-based models differ in their scale and mechanistic assumptions, giving divergent predictions of fire drivers and extent. Although humans have historically used and managed fires, the current role of anthropogenic drivers of fires remains less quantified. Whereas patterns in fire–climate interactions are consistent across the globe, fire–human–vegetation relationships vary strongly by region. Taking a data-driven approach, we use an artificial neural network to learn region-specific relationships between fire and its socio-environmental drivers across the globe. As a result, our models achieve higher predictability as compared to many state-of-the-art fire models, with global spatial correlation of 0.92, monthly temporal correlation of 0.76, interannual correlation of 0.69, and grid-cell level correlation of 0.60, between predicted and observed burned area. Given the current socio-anthropogenic conditions, Equatorial Asia, southern Africa, and Australia show a strong sensitivity of burned area to temperature whereas northern Africa shows a strong negative sensitivity. Overall, forests and shrublands show a stronger sensitivity of burned area to temperature compared to savannas, potentially weakening their status as carbon sinks under future climate-change scenarios.

## Introduction

Fires have been an integral part of the Earth system^1^ since the late Silurian c.420 ma^2^, while hominin-controlled fires have occurred since the Middle Pleistocene c.700 ka^3^. Climate and human activity are thought to be the critical determinants of wildfire frequency, intensity and extent presently^1, 4, 5^. In turn, fires have not only shaped vegetation type at regional scales^6^ but can also cause abrupt shifts in vegetation state^7^. Although c.40% of the land area is fire-prone^6^, an average of c.3% of the land area has burned every year in recent decades, resulting in mean global carbon emissions of 2.2 PgC/yr which is c.25% of global anthropogenic C emissions^8, 9^. Although most natural wildfires are expected to be carbon neutral in the long run, the time required to sequester the burnt biomass may well run into several decades, especially in forest ecosystems^10, 11^. Repeated fires may further hinder sequestration, potentially resulting in positive net carbon emissions. Wildfires also pose serious threats to human safety^12^.

Biomass-burning related greenhouse gas (GHG) as well as non-GHG emissions^13, 14^ and the changed post-burn albedo^15^ alter the atmospheric radiative balance, causing cascading effects on climate and vegetation^1, 10^. Therefore, adequately characterizing the climate–human–vegetation–fire interactions is crucial to projecting the future of the Earth system, especially in the context of increasing human activity and ongoing climate change^16, 17^. Essentially, fires need sufficient fuel (biomass) in a flammable state (low moisture and high density), environmental conditions suitable for enhancing fuel production, flammability, and fire spread, and a source of ignition (lightning, humans)^18^. Studies have suggested that, on the one hand, human influence is causing a decline in global burned area^19^ whereas, on the other hand, increasing global temperatures may lead to an increase in burned area in future^20–22^.

**Figure 1:**
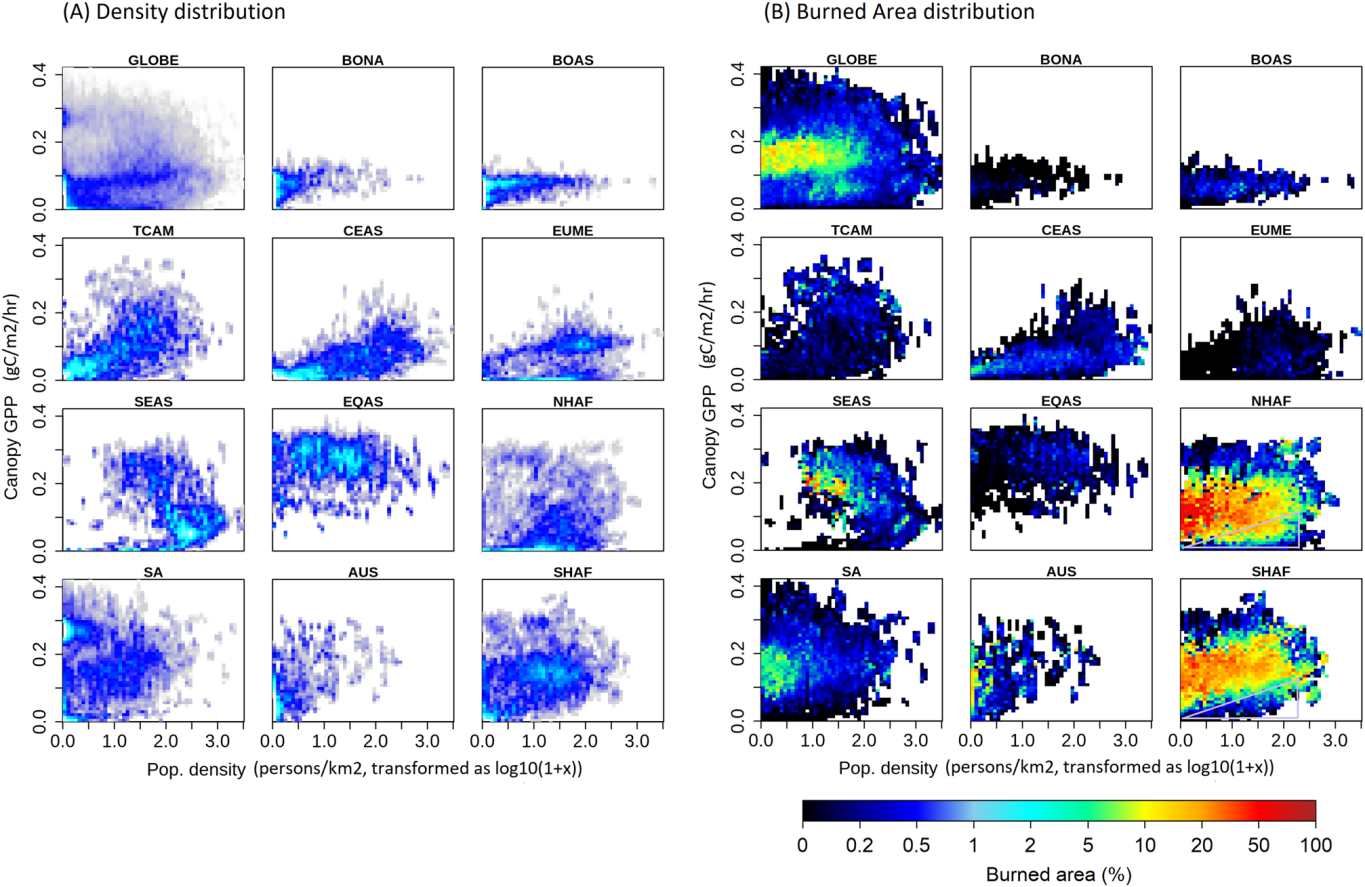
Regional differences in fire regimes can be seen along the GPP-population density axis. The frequency of occurrence of different GPP-population density driver pairs (**A**), and the mean burned area observed for each pair (**B**). Population density is log-transformed with the function ($y=\text {log10}(1+x)$). In general, fires occur at intermediate values of GPP and decrease with population density. However, the responses of burned area to population density are starkly different in different regions: In South America, burned areas are already low at low population densities, and decrease sharply to almost zero at population densities >3 persons/$\hbox {km}^2$. By contrast, fires persist till very high population densities and decline only gradually with increasing population density in Africa. In Australia, burned areas are high at near-zero population densities, but decline sharply even for small population densities.

**Figure 2:**
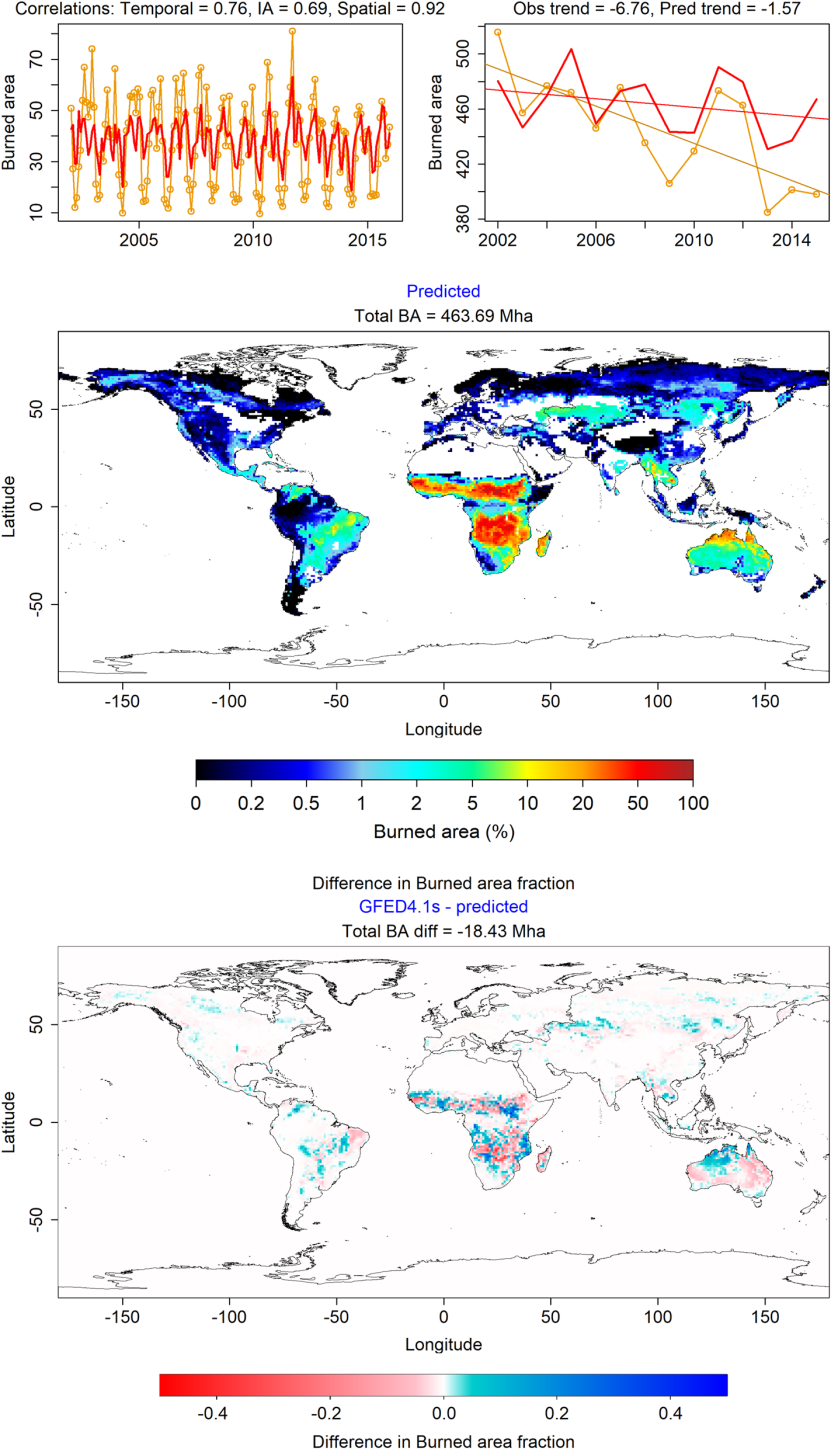
Spatio-temporal performance of our model at the global scale. Temporal monthly (**A**), annual (**B**), and spatial (**C**) burned area predicted by our model (solid red lines) compared with the GFED4.1s burned area data (orange lines and circles). Our model accurately predicts the spatial distribution of fires across the globe, with a spatial correlation of 0.92. It captures the yearly anomalies in global burned area reasonably well (with temporal correlation of 0.76 and interannual correlation of 0.69), and predicts a long term decline of 1.57 Mha/$\hbox {year}^2$ during the years 2002–2015, compared to an observed decline of 6.76 Mha/$\hbox {year}^2$.

The nature of the relationships between fire and its socio-environmental drivers can be conveniently visualized in ‘niche plots’ (Fig. 1, SI-Fig. 2). The fire niche can be thought of as an n-dimensional hyper-volume with positive burned area, in the space of the socio-environmental variables. Climate imposes universal constraints on fires: fires are limited when temperatures are very low, occurring largely at temperatures above $15\,^{\circ }$C. Fires decline beyond temperatures above $30\,^{\circ }$C, which is a result of high-temperatures coinciding with low productivity, and therefore, low fuel availability (Fig. 2). Similarly, high precipitation in the coincident month typically suppresses fires, with most fires occurring when precipitation is below 5 mm/month (Fig. 2). Most fires occur at intermediate values of productivity^23, 24^ across all regions (Fig. 1), but the magnitude of burned area for a given value of productivity differs strongly between regions. Furthermore, strong regional differences can be observed along anthropogenic dimensions, where seemingly similar biomes can have very different fire regimes due to differences in human activity. Burned area declines sharply with population density in Australia and South America, declines gradually and persists until much higher population densities in Africa, and even increases with population density in Boreal and Equatorial Asia. There is also a substantial difference in the fire niches of northern and southern Africa despite similarities in environmental conditions, biomes, and flora: in northern Africa, low GPP areas have high burned areas even with high population densities but not so in southern Africa (marked with a triangle in Fig. 1B). Along most axes, the shape of the fire niche is highly non-linear. The overall magnitude of regional burned area can be understood by superimposing the fire niche on the density distribution of the drivers in the same n-dimensional space (Fig. 1A). For example, southern Africa has high population densities in regions with intermediate GPP, which is absent in northern Africa. Such anthropogenic differences are expected to confound fire–vegetation interactions.

At the global scale, biophysical process-based fire modules have been developed as components of dynamic global vegetation models (DGVMs)^25–28^, which have grown increasingly complex over time. However, despite their complexity and mechanistic appeal, DGVMs have only a modest accuracy in predicting the spatial patterns of burned area, with global spatial correlations between predicted and observed burned area in the range of 0.16–0.69 at a resolution of $1^{\circ }$–$2.5^{\circ }$^29, 30^. Most models are also unable to predict the long-term decline in global burned area over the last two decades^19^, or the interannual variability in burned area, with many DGVMs performing worse than random null models^31^. One reason for the lack of accuracy of global models could be a poor characterization of the human–vegetation niche, which, unlike fire–climate interactions, qualitatively differs between regions. In such a case, a better understanding of the the human drivers of fire could be derived from an empirical framework that does not require any a priori assumptions regarding how humans influence fires.

Empirical approaches have been widely used to predict fire activity and to identify the drivers of fire^19, 32, 33^. However, non-linearities in fire–driver relationships pose a strong constraint on the accuracy of simple regression models. A few studies that have accounted for non-linearities using more advanced statistical analyses^34, 35^ have been limited to specific regions or specific years. Empirical analyses usually also treat the spatial and temporal dimensions of fire separately, i.e. they aggregate one dimension while analysing the other. Such a separation allows for analysis of drivers in space and time, but do not yield predictive models of burned area.

To address these twin issues of regionality and non-linearity, we use a machine-learning framework to understand the specific regional patterns of fire–climate–vegetation–human interactions. We develop a multilayer neural network model to predict burned area from socio-environmental drivers. Previously, studies have employed a similar approach^36–38^ to predict fire incidence probability. Here, we extend this approach to directly predict burned area. As a proof of concept, we test how far a purely data-driven approach can go in predicting burned area. Following the standard paradigm of machine learning, we do not use any hard-coded features, such as fire danger indices or drought codes, as part of the training data. Instead, we let the model learn fire-driver relationships exclusively from data. The machine-learning framework: (a) can account for the high skew in the distribution of global burned area as well as the non-linearities in the fire–driver relationships, (b) is sufficiently scalable to take advantage of large climate and socio-economic datasets which have become available, (c) achieves high predictive accuracy with the least number of input variables, and (d) can inform the parametrization of larger vegetation models. Our results open up further possibilities for improving fire prediction by using more advanced model architectures coupled with the increasing availability of data at higher spatio-temporal resolutions.

## Methods

### Choice of burned-area drivers and datasets

A neural network is essentially a niche model, which not only delineates a volume in the space of drivers where fire occurs, but also predicts a value (in our case, burned area fraction) for each point in the driver space. Our choice of drivers broadly accounts for the classic factors that influence fire^18^: (a) fuel biomass, (b) its flammability, and (c) ignition sources and fire management. A full list of variables used in our model can be found in Table 1.

As a measure of fuel biomass, we use various cumulative measures of gross primary productivity (GPP). Fuel may comprise of litter, canopy, and grass. As a measure of litter biomass in deciduous and savanna vegetation, we calculate total GPP over 1 year covering the previous growing season. Since the growth season begins in spring and leaf-shedding happens in fall (or in winter) across the northern hemisphere, the biomass produced in the growth season becomes available for burning in the following year’s summer. Therefore, litter biomass is roughly equal to the total GPP of the previous calendar year. In the southern hemisphere, the same logic is used, except that the calendar year is shifted by 6 months. As a measure of canopy and grass biomass, we use the cumulative GPP over 1 year up to the previous month. Not all the accumulated GPP will end up as fuel, especially due to differences in biomass allocation to roots, stem, and leaves. We do not explicitly model allocation, rather, indirectly account for allocation differences via vegetation type (as defined by the University of Maryland classification in the MODIS land-cover dataset; see Table 1).

Fuel flammability depends on the intrinsic structural characteristics of the fuel and its moisture content. We use vegetation type to account for the differences in flammability and composition of fuel in different biomes. Moisture content of the fuel is accounted for by environmental variables, specifically, temperature, cloud cover, precipitation, and vapour pressure. Precipitation suppresses fire instantaneously, but over longer timescales, can enhance fuel production, which could increase fire activity in the subsequent dry season in fuel-limited landscapes. Previous studies have used various cumulative effects of precipitation to account for these long-term effects. Here, we only use the instantaneous values of precipitation, which has the effect of increasing fuel moisture and reducing flammability. The long-term effects are captured more directly via fuel proxies as described above. In some regions, like southwestern United States, precipitation may be accompanied with lightning, which may promote, rather than suppress fires. We expect that the neural network would learn to distinguish such events from data on cloud cover and lightning intensity.

Ignition sources are accounted for by human population density, cropland fraction, and lightning frequency. Ideally, ignition frequency would also be influenced by the number of wildland–urban interfaces, but in the absence of a global dataset on such interfaces, we use road-network density as a proxy, of which only a single time snapshot is available. Global monthly lightning data is also not available for the full time period considered in this study. Therefore, we have used an aggregated snapshot with global gridded mean monthly lightning climatology. Furthermore, an examination of pairwise relationships between drivers reveals that lightning frequency is strongly (but non-linearly) related to one or more other drivers, such as precipitation and cloud fraction (SI-Fig. 4). Therefore, these drivers further act as a proxy for lightning in the temporal dimension. Fire management depends on fire prevention and suppression activities by individuals as well as institutionalized mechanisms. Human population density, cropland fraction, and road-network density, apart from being ignition sources, are also expected to have a role in fire suppression.

All driving variables are integrated to a (maximum) temporal resolution of 1 month. We acknowledge that fire spread could be triggered by short-term extreme weather events such as heat waves and wind-bursts, which would be lost in monthly data. However, given the already large volume of data at a monthly scale, and the computational demand for the large number of models we trained, we have made a judicious choice to use a monthly temporal resolution.

Table 1 lists all variables considered, along with the datasets used and any pre-computations performed on raw data.

**Table 1:** Gridded datasets used and their soures.

	Variable	Data source	Spa. Res	Time Res	Details	Citation
**Dynamic variables**						
1	Temperature	CRU TS4.01	$0.5^{\circ }$	Monthly	Mean monthly temperature	^39^
2	Vapour pressure	CRU TS4.01	$0.5^{\circ }$	Monthly	Mean monthly vapour pressure	^39^
3	Cloud cover	MODAL2 / MOD06	$0.05^{\circ }$	Monthly	Mean monthly cloud fraction, coarse-grained	
4	Precipitation	GPCP2.3	$2.5^{\circ }$	Monthly	Monthly total precipitation, transformed as $log(1+x)$	^40^
5	Current GPP	MOD17A1	$0.05^{\circ }$	Monthly	Mean monthly GPP	^41^
6	Growing season GPP	MOD17A1	$0.05^{\circ }$	Monthly	For northern Hemisphere: Sum of monthly GPP over 12 months of the previous calendar year (Jan–Dec). For souhthern hemisphere:	^41^
7	Cummulative GPP	MOD17A1	$0.05^{\circ }$	Monthly	Sum of monthly GPP over 12 months up to the previous month	^41^
8	Population density	GHS GPW4	$0.05^{\circ }$	5 yearly	Population density, transformed as $log(1+x)$, interpolated to monthly from 5-yearly data	^42^
9	Road network density	GRIP4	$0.25^{\circ }$	Snapshot	transformed as $log(1+x)$	^43^
10	Vegetation type fractions	MOD12Q1 / MCD12C1	$0.0083^{\circ }$	Snapshot/yearly	Converted to fractions as described in the main text	^44, 45^
11	Lightning frequency	WWLLN	$0.0083^{\circ }$	Monthly climatology		^46^
**Calibration**						
11	Burned area	GFED4.1s	$0.25^{\circ }$	Monthly		^9^

### Choice of regionalization

Since the human drivers of fire are very distinct across regions, we train separate models for each region. The basic idea behind regionalization is that different grid-cells in the region share certain commonalities. The common features could be environmental, for example, gridcells spanning the same vegetation type, or anthropogenic, such as regions with different levels of human influence. Since the neural network is expected to learn the differences in burned area arising from the input socio-environmental drivers, we wanted the regions to share common features that are not already accounted for by the input drivers. Contiguous geographic regions within the same continent would be expected to have relatively greater uniformity in human features such as management practices, cultural habits, and economic status. Therefore, as a reasonable classification, we use the regions as defined in the Global Fire Emissions Database (Table 2). Furthermore, this also allows us to compare our region-specific results with previous studies. Finally, the spatial delineation of regions must be fine enough to capture such differences, but broad enough to generate sufficient data for training. To ensure that sufficient training data is generated for the models, we have combined regions with low geographical area or fire incidence: TENA + CEAM = TCAM, NHSA + SHSA = SA, EURO + MIDE = EUME.

### Choice of spatial resolution

To unify the temporal and spatial dimensions, we need a spatial resolution at which the negative effect of past fires on current burned area is low. Therefore, we choose a coarse spatial resolution of $1^{\circ } \times 1^{\circ }$. At this scale, as long as burned fractions are low, new fires can still occur in other parts of the gridcell which were not previously exposed to fire, diminishing the overall effect of fire history. Fortunately, this also works for grid-cells with high burned fractions, because such cells are typically located in African savannas, which replenish fuel every year. To verify this reasoning, we built a null model that predicts present burned area only from annual fire history, and found a strong positive correlation (high predictability) between present fire and fire history. This confirms that at this spatial scale, fire history merely reflects the combined effects of other fire drivers without the negative temporal effect.

Vegetation type can change at a much finer spatial scale, especially in areas fragmented by croplands. To account for these fine-scale variations, we calculate the fractional area under each vegetation type in each grid-cell from a high-resolution (MODIS Land Cover) dataset, rather than using a single dominant type at the cell level. The fraction of each vegetation type is then used as an input to the neural network.

All data was coarse-grained to $1^{\circ } \times 1^{\circ }$ resolution (average of all data-points falling within each $1^{\circ } \times 1^{\circ }$ grid-cell). For model training and analysis, we only consider gridcells with at least 30% non-agricultural vegetation coverage.

### The ‘Neural-Fire’ model

We feed the input variables (Table 1) into a dense neural-network (NN) with a single hidden layer consisting of 12 neurons and Exponential Linear Unit (ELU) activation (SI-Fig. 1). ELU is defined as $ELU(x) = \{x, x>0;\ \exp (x)-1, x<0\}$; it grows linearly when *x* is positive, and decays exponentially when *x* is negative. It is this function that enables the neural-network to learn non-linearities. The output layer consists of 25 neurons with softmax activation. Softmax is defined as $\sigma (z_i) = \exp (z_i) / \sum _{i=1}^{N} \exp (z_i)$ and acts as a generalization of the logistic function to multiple dimensions. It is a smooth approximation of the max() function, and normalizes the outputs of the hidden layer of the neural network into a probability distribution over the final output classes. The NN architecture was finalized through trial-and-error: we tried different architectures for the NN, including single-layer architectures with 5–24 neurons, and two-layer (deep) architectures. The performance of the single-layer NNs saturated at about 12 neurons, and deeper architectures did not give any better performance. Therefore, for all our models, we used the single-layer 12-neuron architecture.

To account for the high skew in the burned area distribution, we divide the burned area range [0, 1] into 25 intervals (classes). The first interval is $$[0, 10^{-6})$$ and the remaining 24 intervals divide the range $$[10^{-6},1]$$ equally on a log scale. Each output neuron predicts the probability $$p_i$$ of burned-area being in class *i*, from which we calculate actual burned area as $$BA=\sum _i p_i B_i$$, where $$B_i$$ is the geometric mean of the bounds of class *i*.

We train the model using the GFED4.1s burned area dataset^9^, which specifically accounts for small fires neglected in earlier datasets. We divide our data into training, evaluation and test datasets, and train the network by minimizing cross-entropy on the training dataset. To minimize overfitting, we drop neurons randomly with a dropout rate of 0.05 during training. We halt training when prediction accuracy converges on the validation dataset. We evaluate the performance of different alternative models (i.e., models with different combinations of predictors) on all data, which includes the test dataset. We use monthly data from 14 years between 2002 and 2015 for our analysis. Of these, all data in years 2005–2007 is designated as the test-data. From the remaining data (all grid-cells for all months except 2005–2007), a random sample of 70% of the data points is used for training, and the remaining 30% data points are used for validation. To minimize overfitting, we keep the number of neurons in the hidden layer to a minimum, such that no substantial accuracy is gained from further increasing it. The code to format data and run the Neural-Network model is publicly available at https://github.com/jaideep777/Neural-Fire.

### Measuring model performance

**Figure 3:**
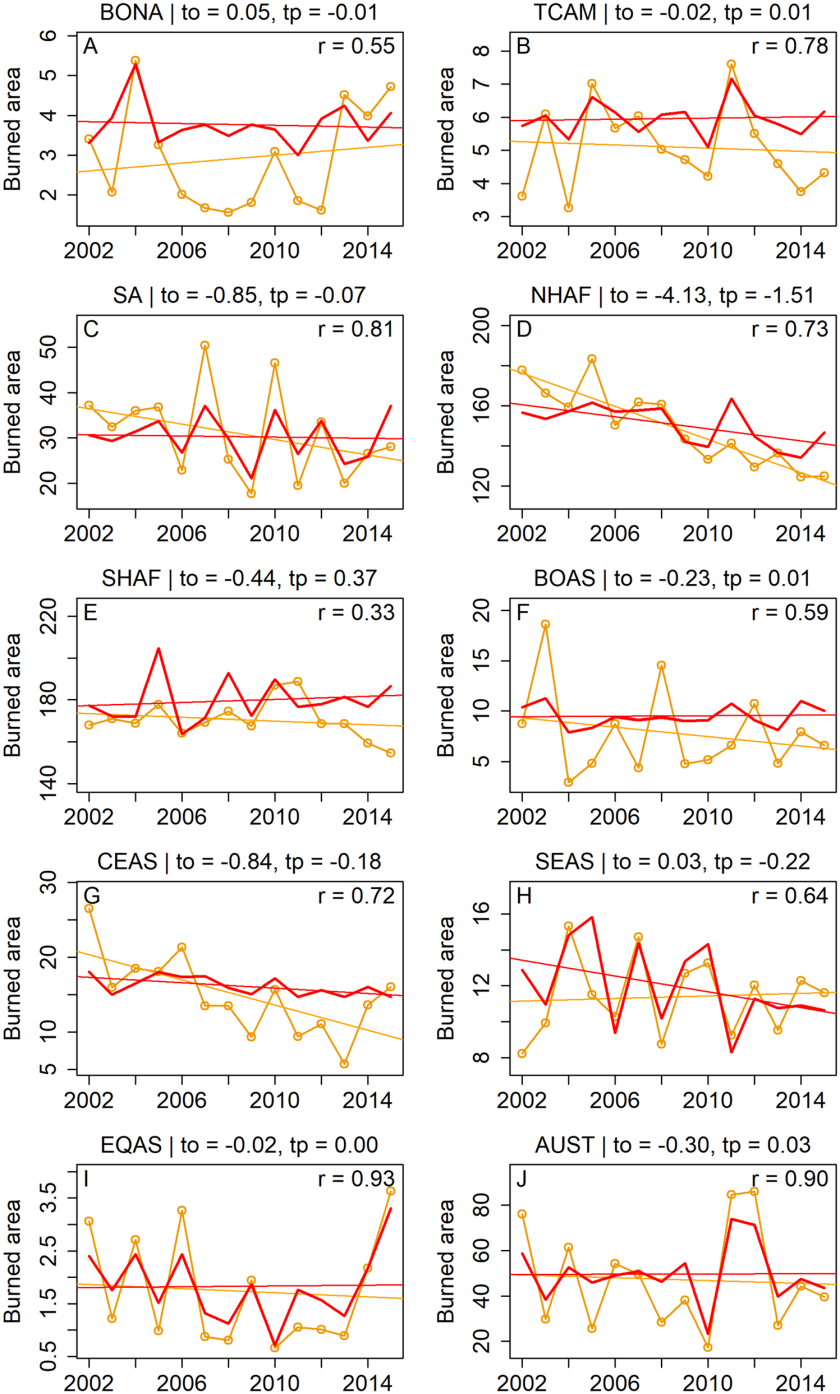
Predicted and observed interannual burned area. Total annual burned area observed in different geographic regions (orange lines and circles) along with that predicted by the minimal model for each region (solid red line). Regression lines indicate the long-term trend in burned area, with the trends in observed ($t_o$) and predicted ($t_p$) burned areas mentioned above each panel. Interannual variability in burned area is well captured in our model, especially in Equatorial Asia, Australia, Southeast Asia, and South America. Long-term decline is highest in northern Africa, with our model predicting 36% of the observed decline. Fires in southern Africa drop sharply after 2013.

To rank models by performance, we calculate five performance metrics for each model—monthly and interannual correlations between spatially aggregated monthly and yearly timeseries of burned area ($r_T$ and $r_{IA}$), correlation between predicted and observed yearly anomalies ($r_{An}$), spatial correlation between mean yearly burned area ($r_S$), and fractional deviation of predicted total yearly burned area from that observed ($r_{BA} = 1 - \text {abs}(1-BA_\text {predicted}/BA_\text {observed})$). We then combine these metrics with weights ($w_i$) into an aggregate performance score $P = 100\sqrt{\frac{\sum {w_i r_i^2}}{4\sum {w_i}}}$, which ranges between 0 and 100, higher the better. These metrics are not used in NN training, but only to rank trained models (i.e., the models are optimized for cell level, and not aggregate, performance). We aim to identify models that have good inter-annual predictability. However, since the spatial extent of data is much greater than its temporal extent, if all weights were equal, models that perform well spatially would receive a higher score even if they delivered poor interannual predictability. Therefore, to privilege models with better interannual predictability, we use $w_{IA} = 4$ and all others weights $w_i=1$. We report the correlation between predicted and observed BA in individual grid-cells ($r_I$, SI-Fig. 3), but do not account for it in evaluating the model performance. This is because we found $r_I$ to be a poor indicator of model performance: we tried a simple linear model using the same set of drivers, and found that it produced $r_I$ values similar to the neural-network model, but predicted incorrect spatial and temporal fire patterns and total burned area.

For each region, we begin with training a model that uses all socio-environmental variables as predictors. Then, we drop one or more variables, trying out different combinations of drivers and measuring the model performance *P*, until we arrive at the best performing model. We then further drop variables to arrive at a ‘minimal model’, i.e., a model that uses the least number of variables without a substantial performance loss compared to the best model (we use the criterion, $P_{best} - P_{minimal} \le 3.0$). For global analysis, we mosaic predictions from the minimal regional models for each month.

## Results

### Model performance

At the global scale, predictions from our model (mosaicked regional models) closely match observations (Fig. 2; see SI-Table 1 for the complete set of models and their regional performance statistics). Our model accurately captures the spatial, seasonal, and interannual variability in burned area, with correlations between predicted and observed data as follows: spatial correlation using temporally averaged burned area—$r_S = 0.92$, temporal correlation using global monthly burned area—$r_T = 0.76$, interannual correlation using global yearly burned area—$r_{IA} = 0.69$, and individual correlation (between burned area of individual gridcells across time and space)—$r_I = 0.6$ (SI-Fig. 3). We also evaluated the performance of our model among different vegetation types, in which $r_I$ varies between 0.31 and 0.78. The model performs best in savannas and broadleaved evergreen forests, and worst in closed shrublands and needleleaved forests (SI-Fig. 3). Further, our model predicts an average annual global burned area of 460.41 Mha/year, against an observed value of 445.26 Mha/year.

Models trained for different regions vary in their performance. The best predictability (performance score $\ge 95$) is achieved for fires in Equatorial Asia, Australia, and South America, whereas those in Boreal regions, Europe, and the Middle East are the least predictable (score between 74 and 85) (Table 2). Model predictions of interannual fire patterns are best in regions with frequent fires ($r_{IA}$ range between 0.68 and 0.89). $r_{IA}$ is lowest in regions with rare fires, especially in Boreal regions (0.56–0.59). Our models suffer from a slight positive bias in regions which contain very high burned fractions, such as in interior Australia and equatorial southern Africa. In such regions, our model predicts a burned area of about 2.5% in cells with extremely low burned area ($< 1\%$).

Figure 3 compares the predicted and observed interannual burned area and long term trends for each region. Although 15 years of data are too short to train our model to capture long-term trends, our model does capture $\sim \,23\%\ (1.57 \text { Mha/year}^2$ of $6.76 \text { Mha/year}^2)$ of the observed global decline in burned area (derived from GFED4.1s data). More than $60\%\ (4.13 \text { Mha/year}^2)$ of the observed global decline is contributed by northern Africa, out of which our model captures $36\%\ (1.51 \text { Mha/year}^2)$ (Fig. 3D) with precipitation, cloud cover, and human population density as the drivers and fixed vegetation type fractions, and $39\%\ (1.60 \text {Mha/year}^2)$ with dynamic vegetation fractions (SI-Table 1). By contrast, only three of seven process-based models from the FireMIP project running at a sub-yearly temporal resolution (CLM fire module, MC-Fire, and JULES-INFERNO) predict a negative trend closer to the actual, but all three substantially underestimate mean global burned area ($< 350$ Mha/year)^19^.

**Table 2:** Regional predictors of fire.

Region code	Region name	Monthly cor.	Interannual cor.	Spatial cor.	Burned area	BA trend	Anomaly cor.	Score		
		${{r_T}}$	${{r_{IA}}}$	${{r_S}}$	BA	LT	$r_{An}$	P	Vars	Variables
NHAF	Northern Hemisphere Africa	0.87	0.77	0.88	151.9	− 1.60	0.53	88.1	3	pr, cld, pop
SHAF	Southern Hemisphere Africa	0.91	0.32	0.92	180.2	0.37	0.36	73.5	3	gppl1, ts, cld
SA	South America	0.92	0.89	0.85	30.7	− 0.17	0.91	94.8	7	gpp, gppm1s, pr, ts, cld, pop, rdtot
SA		0.91	0.81	0.79	30.3	− 0.07	0.85	91.9	5	gpp, gppm1s, pr, ts, cld
SEAS	South and Southeast Asia	0.89	0.68	0.87	11.8	− 0.19	0.77	87.7	8	gpp, gppm1, pr, ts, cld, vp, pop, rdtot
SEAS		0.88	0.64	0.89	12.0	− 0.22	0.73	86.0	6	gpp, gppm1, pr, ts, cld, pop
TCAM	Temperate and Central America	0.77	0.84	0.74	6.3	− 0.01	0.84	90.7	5	gpp, gppl1, pr, ts, cld
TCAM		0.77	0.78	0.73	6.0	0.01	0.79	89.1	4	gpp, gppl1, pr, ts
BONA	Boreal North America	0.83	0.56	0.67	3.3	0.00	0.57	81.2	6	gpp, gppl1, pr, ts, cld, vp
BONA		0.83	0.55	0.56	3.8	− 0.01	0.57	79.4	3	gpp, gppl1, pr
AUS	Australia	0.86	0.91	0.91	50.2	0.21	0.92	95.1	6	gppm1s, gpp, gppl1, ts, cld, vp
AUS		0.86	0.90	0.88	50.5	0.03	0.90	94.6	3	gpp, gppl1, cld
CEAS	Central Asia	0.70	0.72	0.79	16.2	− 0.18	0.55	85.5	4	gppl1, pr, cld, vp
BOAS	Boreal Asia	0.65	0.59	0.82	9.5	0.01	0.62	82.2	4	gppm1, pr, ts, vp
EQAS	Equatorial Asia	0.80	0.93	0.87	2.0	0.02	0.95	95.0	3	pr, ts, cld
EQAS		0.76	0.93	0.78	1.9	0.00	0.94	93.7	1	pr
EUME	Europe and Middle East	0.83	0.34	0.67	2.6	0.00	0.35	70.7	2	pr, cld

Performance of the best and minimal models for each region with respect to each of the five performance measures described in Methods, along with the aggregate performance score. In some regions, the best model is the same as the minimal model. Also mentioned are the variables that form the inputs of the models. BA is Burned Area, and LT is long-term trend in spatially aggregated yearly timeseries. Variables are as follows: gppl1—cumulative GPP, gppm1—growing season GPP (northern hemisphere), gppm1s—growing season GPP (southern hemisphere), pr—precipitation, ts—temperature, cld—cloud cover, vp—vapour pressure, rdtot—total road network density, pop—population density. All models include vegetation type fractions, including cropland fraction. The model for NHAF uses yearly vegetation fractions, whereas rest of the models use a single snapshot.

### Sufficient regional predictors of fire

For each region, we obtain the sufficient regional predictors of fire from the inputs of the regional minimal models. Regional sufficiency of socio-environmental factors does not necessarily imply that other factors are not of mechanistic importance in fire ignition and spread. A factor that clearly favours fire may drop out of a regional model if, (a) it is not sufficiently variable in the subcontinental region (e.g., fuel load is always high in Equatorial Asia, and population density is always very low in Australia), or (b) if it is correlated with another factor that influences fire (e.g. in Australia, either of temperature and cloud cover is sufficient to predict fire because both are correlated, but temperature drops out of the minimal model for Australia). On the contrary, factors that are not necessarily limiting (such as fuel load in Boreal regions) may still be significant predictors due to variability within the region. The sufficient predictors identified here should thus be interpreted as those which have the highest predictive value at the subcontinental scale, given the combined effects of all socio-environmental drivers.

Within each region, climate and fuel load explain the spatio-temporal patterns of fire for all regions except northern Africa and Southeast Asia, where human population density is additionally a significant predictor (Table 2). Fuel load turned out to be a significant predictor in all Boreal regions in our study, whereas it was not considered to be significant in previous studies (SI-Table 3). Vegetation type fractions explain most of the spatial variability in fires across all regions, whereas climate and fuel load were the most important predictors of seasonal and interannual variability. Among the anthropogenic factors considered, population density had a negative effect on fire, with a monotonic decline in burned area with increasing population density, but within-region variability in population density was important only in northern Africa and Southeast Asia.

To test the effect of cropland fraction, we excluded two vegetation type fractions from model training (fraction of area under croplands as well as the fraction of non-vegetated area from the minimal model for each region), and compared the resultant models with the original minimal models. For this analysis, it is not enough to exclude only cropland fraction: as vegetation-type fractions add up to one, excluding any one fraction still provides the neural network with all land cover information. Cropland fraction was a significant predictor in Southeast Asia and Boreal North America (i.e. predictability reduced when cropland fraction was excluded in these regions). In Boreal Asia and Central Asia, exclusion of cropland fraction improved predictability, implying that cropland fraction is neither a consistent driver nor a consistent deterrent of fires in these regions.

Road network density and lightning climatology showed no substantial explanatory power within regions, and dropped out of all regional minimal models (for the effects of lightning, compare version 8 models in SI-Table 1). However, data on both these variables were not available for multiple years. Therefore we do not rule out their effect on fires based on this study. Specifically, including monthly lightning data may improve predictions in Boreal regions, as these regions are known to be ignited by lightning in recent years^47^.

Droughts associated with El Niño events have been shown to strongly influence fires across the tropics, especially South America and Equatorial Asia^48^. Higher fires associated with El Niño events are observed in South America in the years 2007, 2010, and 2015 (Fig. 3C)^49^, and in Equatorial Asia in 2002, 2004, 2006, 2009, and 2015 (Fig. 3I)^50^. Our model correctly predicts high burned area in these regions and years. Furthermore, the extreme fire events observed in Australia in 2011 and 2012 appear to be caused by negative values of the Interdecadal Pacific Oscillation (IPO) coupled with El Niño (negative values of the Southern Oscillation Index)^51, 52^. Our model also predicted high burned areas in Australia during these years (Fig. 3J). Therefore, our model could potentially be used to forecast the next El Niño-driven fire season for these regions.

Our models were also able to broadly distinguish fuel characteristics in different regions. In Africa, Australia, and Central Asia, the cumulative GPP up to the previous month (a proxy for grass biomass) featured in the minimal models, whereas in Southeast Asia the previous calendar year’s GPP (a proxy for litter biomass) did. In South America and Australia, both litter and canopy biomass were equally good predictors of burned area, implying that both might constitute the fuel in those savannas.

### Climate sensitivity

**Figure 4:**
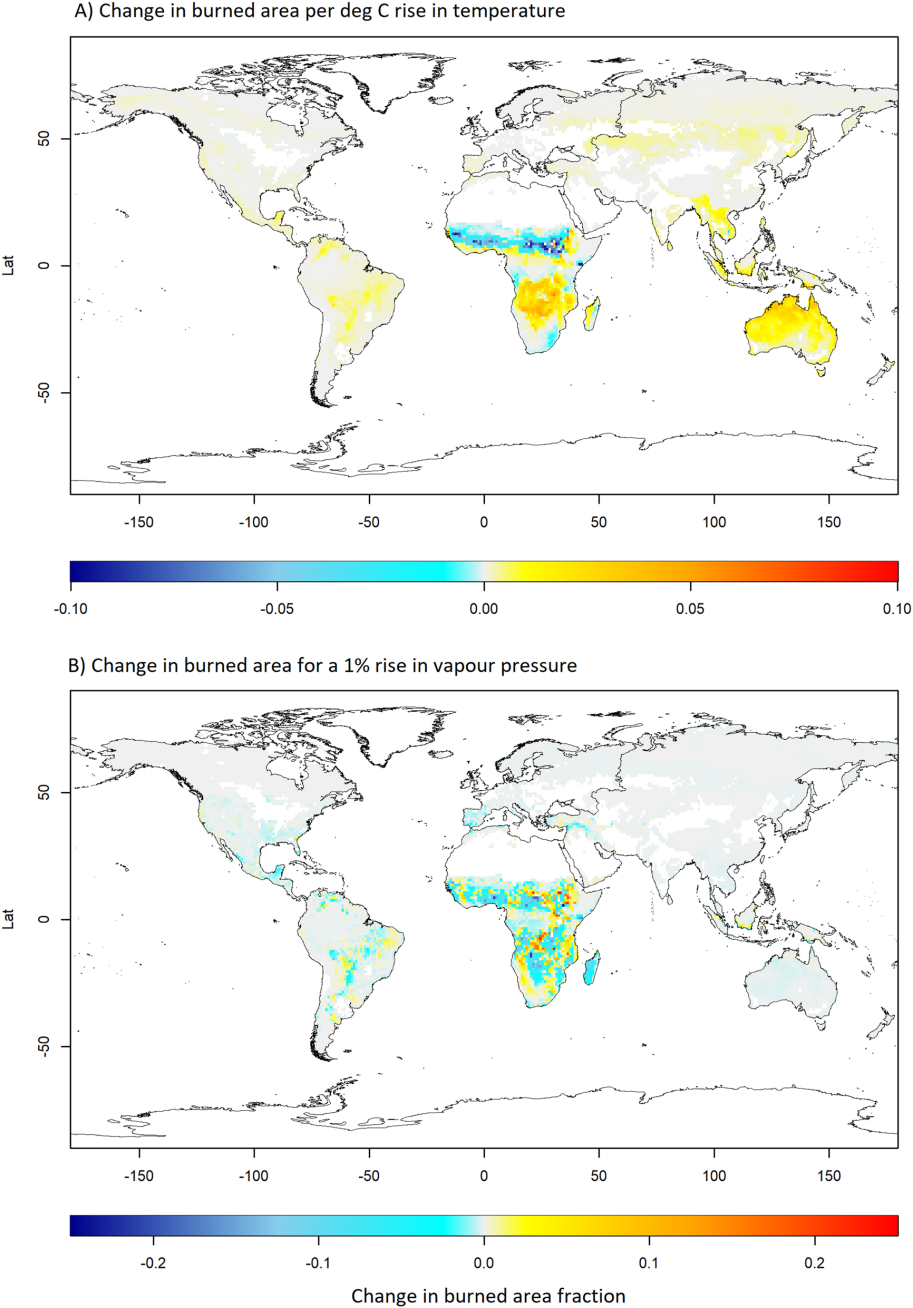
Sensitivity of burned area to temperature and vapour pressure. Change in burned area fraction per $^{\circ }$C rise in temperature (**A**) and for a 1% increase in vapour pressure (**B**). While incrementing either variable, all other variables were held equal to their original values.

Given the current socio-environmental conditions, how will different regions respond in terms of wildfire vulnerability to increasing global temperatures? Towards answering this question, we drive the best regional model for each region with the same input data from the time period 2002–2015, but with temperature uniformly incremented by a small amount ($\Delta T$), while keeping other variables at their original values. This small change in temperature is assumed to have no effect on the vegetation type distribution. In case temperature has dropped out of the best regional model, we choose the next best model that includes it (SI-Table 2 lists the models used in such cases). We then measure the sensitivity of burned area to temperature as the change in burned area fraction per unit change in temperature ($S = \Delta BA / \Delta T$), and as percent change per unit temperature ($S_\% = \Delta BA /BA /\Delta T$).

Globally, forest-dominated areas show the highest sensitivity 10.46–18.75%/$^{\circ }$C of burned area to temperature (Table 3). Grasslands and croplands show moderate sensitivity ($4.91\%/ ^{\circ }$C and $8.65\%/ ^{\circ }$C respectively), whereas savannas show a negative slight sensitivity ($-0.57 \%/ ^{\circ }$C). In absolute terms, the most sensitive areas are concentrated in Equatorial Asia, southern Africa, and northern Australia (Fig. 4A). This is a result of high sensitivity during the months of August–November (Winter–Spring), when fires are currently temperature limited. Northern African savannas show a strong negative sensitivity to temperature (Fig. 4A), with the effect being strongest in the months of February–April (summer) and weakest in December–January (winter), with some areas even showing a positive sensitivity in winter. Therefore, this decrease is likely due to a reduction in biomass density associated with an increase in aridity (vegetation type is held constant). Southeastern Australia and eastern Himalayan regions have relatively less fires, but are highly sensitive to temperature changes in terms of percent change in burned area (SI-Fig. 8). SI-Fig 5 (animated gif) shows the global sensitivity for each month.

We also performed a similar analysis by incrementing vapour pressure by 1% (Fig. 4B). As expected, an increase in vapour pressure leads to a decline in burned area in most regions. In absolute terms, Africa is again the most strongly affected region, but there is strong spatial heterogeneity in the sensitivity at local scales. However, we note a caveat here that vapour pressure was not a good predictor of burned area in most regions, and thus we could not use the best performing model for this analysis.

**Table 3:** Sensitivity of different vegetation types to increase in temperature.

Ncells	BA (Mha)	Sensitivity ($\Delta$BA(%)/$^{\circ }$C)	Vegetation type
52586	3.39	13.36	Non-vegetated
860	2.29	12.31	Needleleaved evergreen
1306	26.50	15.67	Broadleaved evergreen
232	1.61	10.46	Needleleaved deciduous
82	0.33	12.49	Broadleaved deciduous
906	4.17	14.27	Mixed forests
3562	39.76	18.75	Open shrublands
7	0.02	− 1.68	Closed shrublands
473	129.49	0.62	Woody Savannas
846	171.33	− 0.57	Savannas
1123	21.49	4.91	Grasslands
1553	6.25	8.65	Croplands
1264	56.53	4.37	Mixed vegetation
**64800**	**463.16**	**4.00**	

Compared to a decrease in savannas of 0.57%, there is a disproportionately positive sensitivity of burned area to temperature in forests and open shrublands. Numbers in bold indicate global totals.

## Discussion

We have shown that our machine-learning model delivers high predictive accuracy with only a few input variables. We found distinct regional differences in fire drivers across regions, but within regions, between 1 and 5 drivers are sufficient to accurately predict burned area. We found that whereas climatic constraints on fires were universal, differences in anthropogenic niches may drive regional differences in fire activity. We predicted differential effects of increasing temperature in different regions, with forests being disproportionately sensitive to temperature changes compared to savannas, although we have not accounted for changes in co-varying drivers in this analysis. Our work suggests that an improvement in predictive accuracy of fire models can result from better parameterization of models with fewer drivers, rather than expanding already complex models with more processes and parameters.

Modelling approaches based in machine learning often face the criticism that they do not provide any understanding of the underlying mechanisms and processes. However, as we demonstrate in this work, it is now possible (due to advantages in computational power) to scale up neural-network models and run them iteratively to perform an analysis of the minimal predictors of fire. Such an analysis provides vital information on the relative importance of different drivers in different environmental conditions. Furthermore, it is possible to look into the functional relationships between fire and the most important drivers learned by the model, to make inferences about the underlying mechanisms as well as to parametrize process-based models. Although neural-network approaches have been previously used for fire incidence prediction^36–38, 53, 54^, to our knowledge, our model is the first to predict continuous burned area at continental and global scales. Here, we have used a simple multilayer neural network to demonstrate the effectiveness of a machine learning approach. Our results were robust to different dropout rates and fractions of data used for training (within reasonable limits), and using a deeper NN architecture (with two hidden layers) did not perform any better than one with a single hidden layer (SI-Table 4). However, more advanced architectures could be used in further studies to incorporate specific features of fire, such as Convolutional Neural Networks (CNNs) to capture spatial interactions among grid-cells, or Recurrent Neural Networks (RNNs) which can account for previous fire history. Furthermore, we expect that with increasing availability of high-resolution datasets, the predictive power of neural-network approaches will further increase. Thus, advanced network architectures, coupled with higher resolution data at a daily time-scale, could substantially advance our understanding of fire intensity and spread under different socio-environmental conditions.

Our model does predict cell-level extreme burned fractions with good accuracy (lesser spread towards higher burned fractions in SI-Fig. 3), but fails to distinguish fire extremes at an annual regional scale. For example, the extreme fires in Boreal Asia in 2003, 2007, and 2010 are not captured, whereas high burned area is predicted in Boreal America even in years with low fire activity (2006–2012). A factor responsible for extreme Boreal fires in recent times seems to be short-interval re-burning of previously burned patches, which was rare in the past, but more likely in today’s changed climate^55^. Fire history is not accounted for in our model, which might explain the lack of predictability of boreal fire extremes. Fires during peak years in Equatorial Asia and South America are also slightly underestimated. In general, low predictability of extreme fires might also be due to the rarity of extreme events, such that most of the training data consists of non-extreme fires, and an imbalance in the spatial and temporal extents of the training data. Due to the flexibility of the neural network approach, it is possible to assess the drivers of extreme fire events by training a model with a subset of data containing a greater proportion of extreme burned fractions^56, 57^. A related problem is that long-term annual decline is not fully captured by the models. To mitigate these issues, future work could use data resampling to equalize the spatial and temporal extent of training data.

Studies differ on the predicted drivers of fire for the same regions (SI-Table 1). For example, the drivers of fire in northern Africa are predicted to be precipitation, population density, and cropland fraction^58^, or population density, temperature, and wet days^35^. We find precipitation and population density to be important drivers, but no effect of cropland fraction. In southern Africa, predicted drivers are fuel and climate^58^, or wet days and cropland fraction^35^, or tree-cover, rainfall, dry season, and grazing^34^. We, too, find fuel and climate to be the key drivers. While other studies predict fuel and climate to be the drivers in Equatorial Asia^33^, we found that precipitation alone explained the variability in fires in this region. In Boreal areas, we find an important effect of fuel load, which is not predicted by previous models, but consistent with observations^59^. In Southeast Asia, Abatzoglou et al.^33^ find aridity alone as a driver, whereas we find climate, fuel, as well as population density to be important drivers. However, this difference could also be due to differences in the spatial discretization, where we have used a rectangular grid, whereas Abatzoglou et al.^33^ have used ‘ecoregions’. Indeed, in our model too, using ecoregions instead of grid-cells could improve model performance by reducing the spatial expanse of data, so that temporal trends are better captured, and by reducing the dimensionality of the vegetation type fractions, so that the number of predictor variables is reduced.

Previous studies have attributed the long-term decline in fire in northern Africa to cropland expansion^58^. However, we find that this decline is instead explained most strongly (39%) by increasing population density. We found no performance drop after excluding cropland fraction from the model (compare version 6 models in SI-Table 1), implying a low predictive value of cropland expansion. The residual long-term decline in northern Africa does not appear to be driven by changes in other vegetation types either—we achieved only marginally better predictive ability (39% vs 36%) after including dynamic (yearly) vegetation fractions in our training (SI-Table 1), even though trends in certain vegetation type fractions are weakly correlated with trends in burned area (SI-Figs. 6, 7). This is largely corroborated by a recent study by Zubkova et al.^60^, who also do not find cropland expansion to be the driver of fire in northern Africa; they, however, attribute the changes in burned area to changes in moisture. In our study, a model driven by climate alone predicted only a modest long-term trend, whereas a model including population density predicted a much larger decline. This difference could be at least partially attributed to the differences in the nature and quality of the input drivers—their model uses mechanistically derived values of soil moisture and a high-resolution precipitation dataset, which are expected to be more accurate compared to the global precipitation dataset that was available to us. Furthermore, their model does not include population density as a driver.

Under future climate scenarios, such as with a 2$\times$ or 3$\times$ increase in CO2 concentrations, a complex interplay between temperature and precipitation changes is expected to lead to increased fire activity in some parts of the globe and a decrease in other parts^4^. We expect our model to have ‘learnt’ how temperature-precipitation interactions affect fire. Therefore, in our sensitivity analysis, increasing temperature will be ‘seen’ by the model together with correlated changes in other variables that are not directly input. Therefore, we compare our sensitivity predictions with a maxEnt-based model of fire driven with projections of 16 different climate models^61^ (hereafter referred as ‘other models’). We highlight particularly, the predictions which are counter-intuitive or not corroborated by other models. First, the contrast in fire sensitivity to increasing temperature between northern and southern Africa may seem surprising due to similarities in weather and vegetation. However, other models agree that fires in northern Africa may decline by end of the century, whereas model agreement is low for southern Africa both in historical^31, 62^ as well as projected climates. As we have argued, this could be the effect of differences in the anthropogenic niche of fires in the two regions, resulting in a seemingly anomalous occurrence of fire at low temperatures and high precipitation in southern Africa, compared to the overall trend within SHAF as well as the global temperature-precipitation niche of fires. It could also be due to differences in livestock density, which we have not accounted for in this study. To further confirm the anthropogenic decline in fires in northern Africa, we ran the sensitivity analysis with models including and excluding human population density, where no decline was seen in models without human density. Second, other models also show scarce agreement on the change in fires in southeastern Australia, where our model predicts a high temperature sensitivity (in terms of percentage change). Qualitatively, our prediction may be corroborated by the recent occurrences of large-scale fires in this region during December 2019 to January 2020. Third, a small predicted increase in fires in already-arid interior Australia is also surprising, but is consistent with the consensus of other models. However, there is poor agreement in model predictions for historical periods in interior as well as southeastern Australia^31^, and our result here could also be an artefact of data limitations as we argue below. Fourth, one region where our model disagrees with the consensus of other models is northern Australia, where we predict an increase in winter-spring fires with temperature whereas other models predict and agree on a decrease^61^. However, this discrepancy could likely be explained by accounting for precipitation, which is expected to increase in this region, but has dropped out of our model for Australia. A quantitative analysis of changes in burned area using future projected climatic drivers would provide more accurate projections of fire activity under future climate change scenarios. Our neural-network model can be directly integrated into vegetation models for such analyses.

We caution readers in interpreting the sensitivity values in arid areas in interior Australia and parts of South America. Although we expect fires to reduce at extremely high temperatures due to declines in vegetation cover, in Australia and South America, data-points which show a reduction in burned area at higher temperatures are limited (Fig. 2). Therefore, the neural network does not have the opportunity to learn this declining trend. This is in contrast with other regions which do show a decline in fires for extremely high temperatures. Therefore, the models might overestimate sensitivity to temperature in very arid areas within these regions. This problem could be overcome as more data becomes available.

Fuel consumed in fires and subsequent emissions vary by region^9, 63^. In savanna-dominated Africa, fuel consumption per unit area burned is low, and fires are carbon neutral^10, 11^ even in the short-term, as most of the grass biomass is regenerated the next year. By contrast, in tropical and Boreal forests, fuel consumption is high, not only from standing vegetation, but also from soil carbon, and recovery of lost biomass may take decades^63^. On the one hand, burned area has declined due to human influence in African savannas, leading to an overall decline in global burned area^58^. On the other hand, fires in Boreal and tropical forests are driven by climate, potentially putting these regions at a heightened fire risk due to future climate change^55^. An increase in burned area in these forests may further increase fire related emissions, weakening their status as carbon sinks, and creating a cascading effect on the global climate system.

Our model does not attempt to capture the complex feedbacks from fire into the climate systems^64^ which, in any case, is a limitation even in advanced DGVMs^17^. Neural network models have the potential to be coupled with DGVMs and socio-economic drivers, and may provide a simpler class of models to predict future fire regimes, assess impacts such as GHG and non-GHG emissions, distribution of vegetation types, and risks to society, both at regional and global scales.

## Supplementary Information

Supplementary material 1

Supplementary material 2

Supplementary material 3
