# Supplementary material for: Improving prediction and assessment of global fires using multilayer neural networks
Source: Sci Rep. 2021 Feb 8;11:3295. doi: 10.1038/s41598-021-81233-4 (PMC7870964; doi:10.1038/s41598-021-81233-4)
Supplement: Supplementary file 1 — Supplementary material 1 [file 41598_2021_81233_MOESM1_ESM.pdf]

Supplementary Information: Improving prediction and assessment of global fires using multilayer neural networks

Jaideep Joshi[1,2*], Raman Sukumar[1,2*]

[1] Centre for Ecological Sciences, Indian Institute of Science, Bengaluru, 560012, India.

[2] Divecha Centre for Climate Change, Indian Institute of Science, Bengaluru, 560012, India.

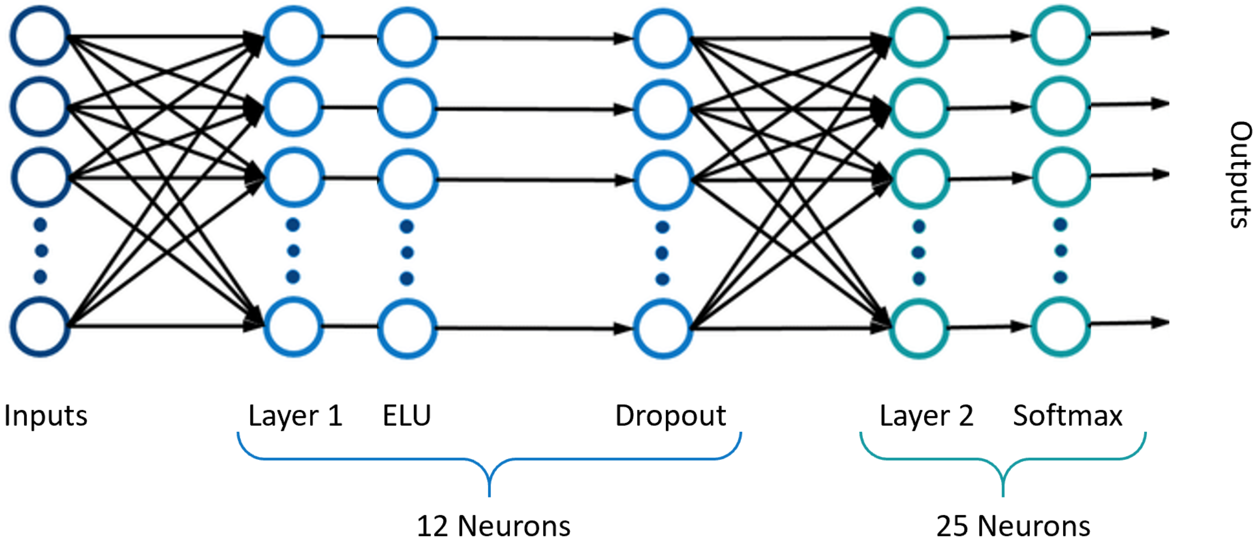

SI-Figure 1. Schematic of the neural network model used in this study.

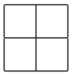

SI-Table 1. Please see SI-Table 1 excel sheet in Supporting Information.

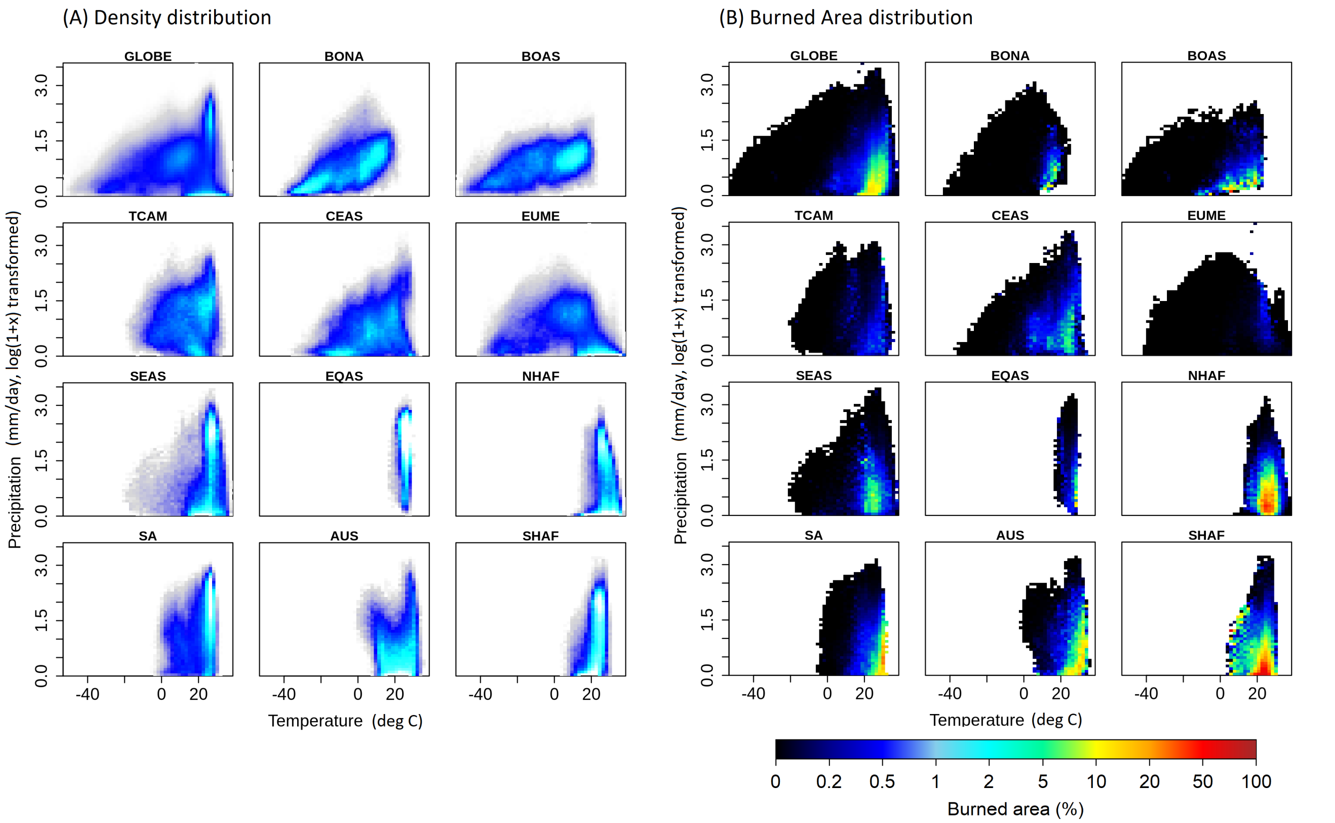

SI-Figure 2. Visualization of the fire niche along the temperature-precipitation axes. A) Each coloured (non-white) point represents a realized value of the driver pair, with the colour indicating mean burned area fraction observed for that driver pair. Here the precipitation axis is log transformed with the function $y = \log(1+x)$. Fires are largely confined between $15-30^{o}$C temperature and < 5 mm/month precipitation (about 1.5 units on the log transformed scale shown here). Notable outliers can be seen in southern hemisphere Africa, where fires are observed at lower temperature and higher precipitation.

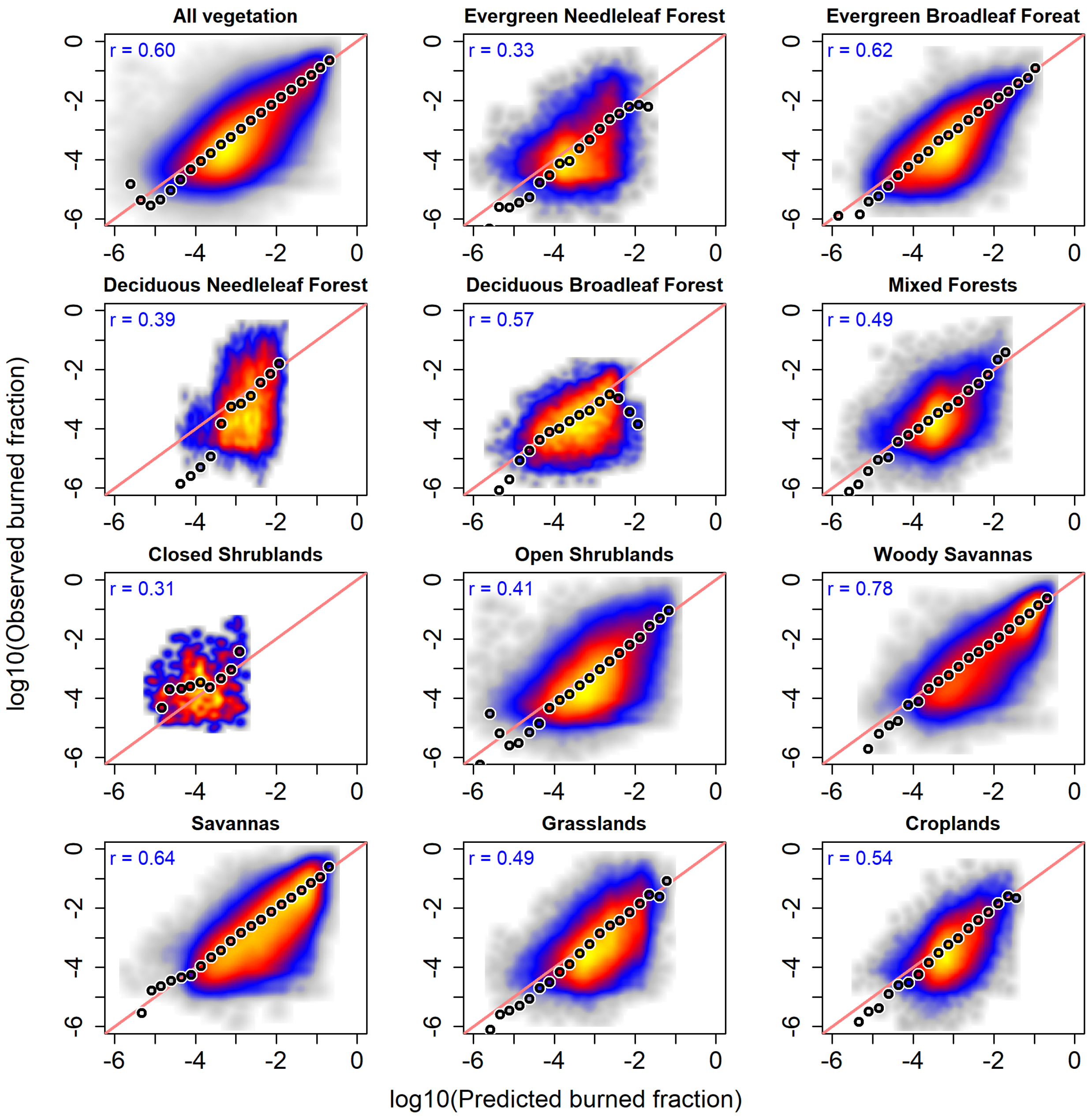

SI-Figure 3. Observed vs predicted burned area for each forest type. Red line is the 1:1 line, and black/white circles show mean BA in each class. Density of points increases from grey to blue to red to yellow. Correlation is indicated in the top left corner. To identify the dominant PFT in each grid, we first excluded all grids with more than 50% non-vegetated or agricultural area (they were classified as non-vegetated and croplands respectively). Among the remaining grids, we ranked the types by abundance. If the most abundant type was at least 10% more abundant than the second most abundant one, we classified the grid as dominated by that type, or else as mixed vegetation.

Region	T	IA	S	BA	LT	IA (det)	Score	Drop	Vars	Variables
NHAF	0.93	0.63	0.92	151.6	-1.02	0.37	82.7	-5.33	4	pr, ts, cld, pop
CEAS	0.71	0.59	0.79	15.4	-0.07	0.57	82.2	-3.34	5	gppl1, pr, ts, cld, vp
EQAS	0.82	0.92	0.83	2.1	0.03	0.94	94.4	-0.54	4	gppm1, pr, ts, cld
EUME	0.81	0.29	0.65	2.2	0.02	0.34	70.5	-0.19	2	ts, cld

SI-Table 2. Models used for sensitivity analysis, in the case where the best regional model from Table 2 does not include temperature. These models may have a substantial performance loss (Score difference > 5) compared to the best model.

	**This study**	**Abatzoglou et al. 2018** [33]	**Andela et al. 2014** [58]	**Ponomarev et al. 2016**[65]	**Werf et al . 2008**[66]	**Aldersley et al. 2011**[35]	**Archibald et al. 2009**[34]
BONA	moisture, fuel	aridity, climate					
TCAM	climate, moisture, fuel	aridity					
SA	climate, moisture, fuel	aridity			fdp		
EUME	moisture						
NHAF	moisture, pop		pr, pop, crop		no fuel, no aridity,	pop, wet, temp	
SHAF	climate, fuel	fuel, climate	pr		no fuel, no aridity,	wet, crop	treecover, rainfall, dryseason, grazing
BOAS	climate, moisture, fuel	aridity, climate		aridity, temp		wet, tmp, crop, precip	
CEAS	climate, fuel						
SEAS	climate, moisture, fuel, pop	aridity					
EQAS	moisture	fuel, climate					
AUS	moisture, fuel	aridity, fuel, climate			npp	tree, precip	

SI-Table 3. Fire drivers in different regions as per our study and key previous studies.

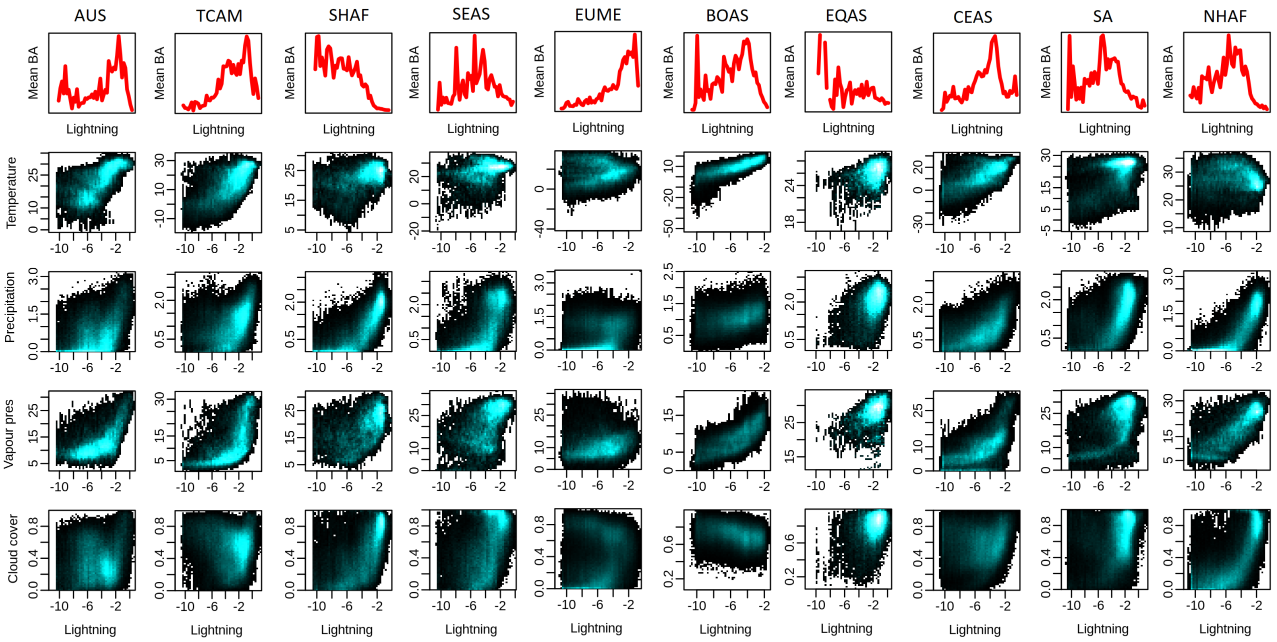

SI-Figure 4. Relationships between lightning and other drivers

SI-Figure 5. Global temperature sensitivity for all months (GIF uploaded separately)

	Performance metrics					Training results			
Notes	r_T	r_{IA}	r_S	BA	P	CE	A(Train)	A(Eval)	A(test)
d = 0	0.85	0.88	0.91	50.5	0.94	1.51	0.60	0.60	0.61
d = 0.05	0.84	0.89	0.92	48.9	0.94	1.55	0.60	0.60	0.61
d = 0.1	0.83	0.87	0.91	47.7	0.94	1.54	0.60	0.60	0.61
d = 0.05, L=2	0.85	0.87	0.91	47.6	0.94	1.52	0.60	0.60	0.61
f = 70%	0.84	0.89	0.92	48.9	0.94	1.55	0.60	0.60	0.61
f = 50%	0.84	0.86	0.90	49.4	0.93	1.53	0.60	0.60	0.61
f = 20%	0.83	0.87	0.91	49.6	0.94	1.51	0.60	0.60	0.61
f = 1%	0.79	0.77	0.84	50.8	0.89	1.32	0.63	0.58	0.59
f = 0.5%	0.75	0.87	0.79	37.2	0.92	1.08	0.68	0.53	0.55

SI-Table 4. Performance of different NN architectures and training data fractions. For this analysis, we used the minimal model for Australia. The models are robust for changes in dropout rates, implying that there is no overfitting. Within reasonable limits, the models are also robust to variations in the fraction of data used for training. Here, CE is the cross entropy on the training dataset, and A is the classification accuracy, reported on training, evaluation, and test datasets, d is the dropout rate, L is the number of hidden layers, and f is the fraction of data used for training.

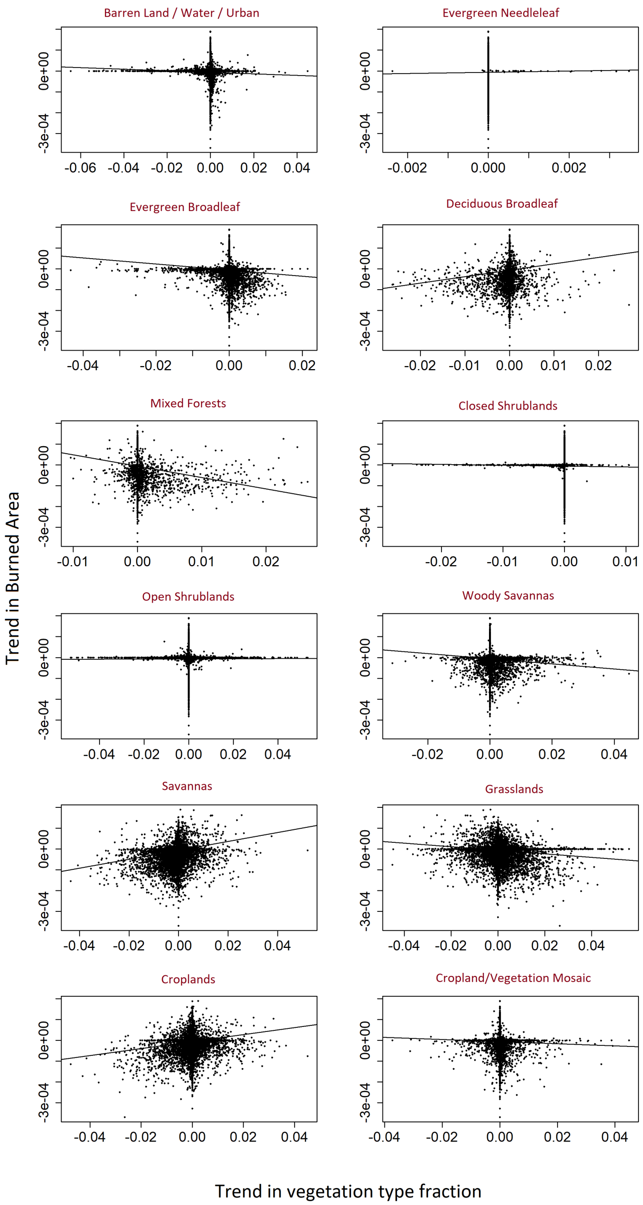

SI-Figure 6. Correlation between trends in BA and trends in each vegetation type

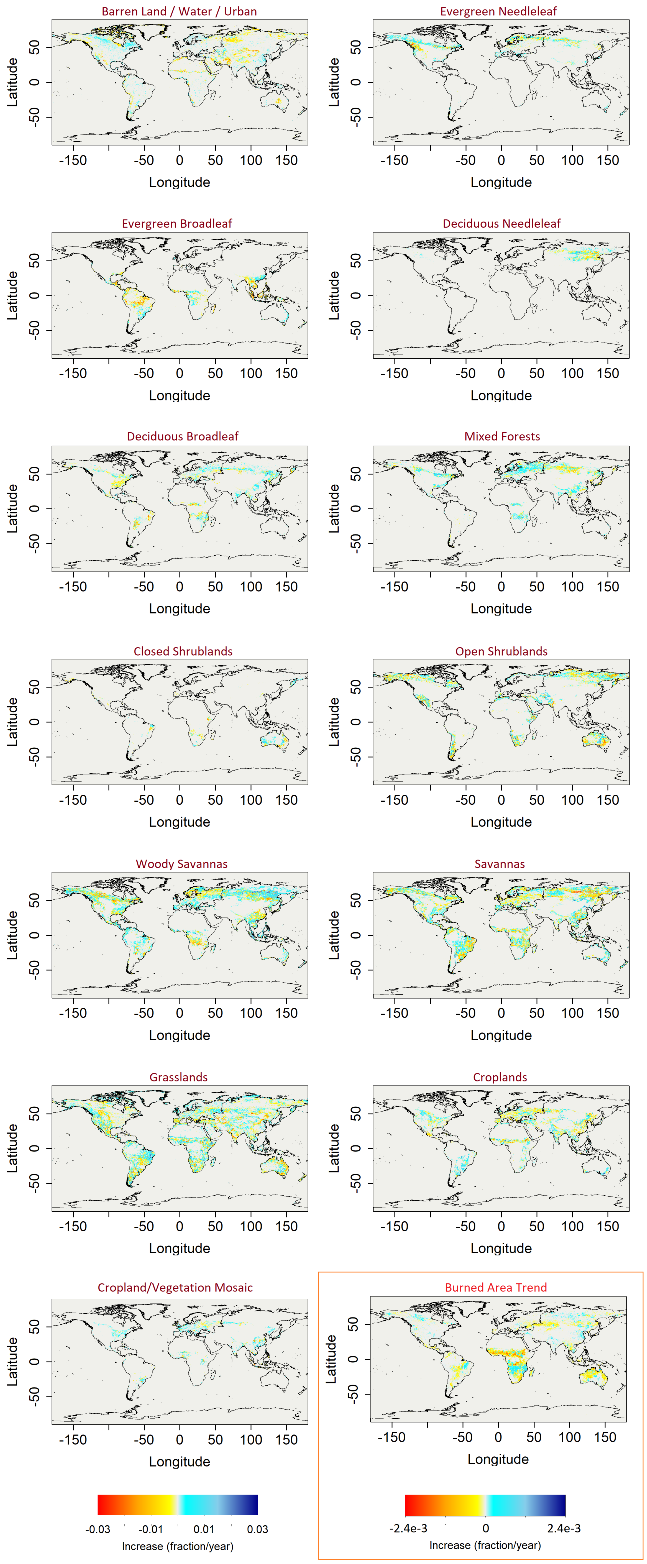

SI-Figure 7. Trends in each vegetation type across the world from 2001-2017, along with the trend in burned area from 2001-2016. Also provided separately as a high-resolution file in SI.

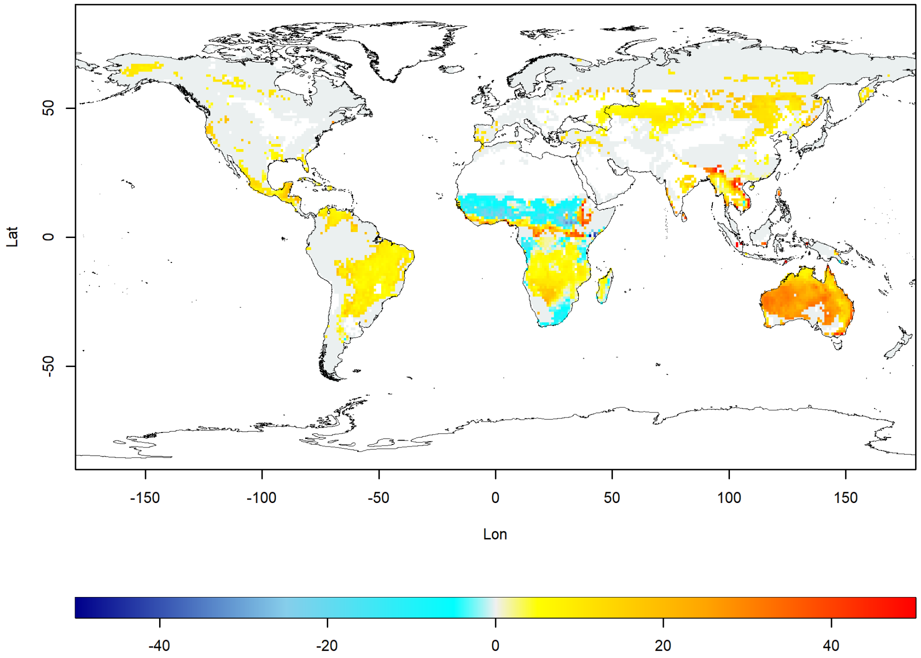

SI-Figure 8. Sensitivity of fire to temperature (percent increase in burned fraction per unit change in temperature). Regions in eastern Himalaya and southeastern Australia stand out in terms of percentage increase in burned area fraction per unit rise in temperature. To avoid spurious values, we have removed cells with extremely low burned areas ($< 1\%$ of the cell burned). The projected high sensitivity of interior Australia is surprising given that temperatures are already high there, but this could be due to a lack of data prescribing a decline at very high temperatures (see discussion).
