# Supplementary figures and images for: Improving prediction and assessment of global fires using multilayer neural networks
Source: Sci Rep. 2021 Feb 8;11:3295. doi: 10.1038/s41598-021-81233-4 (PMC7870964; doi:10.1038/s41598-021-81233-4)

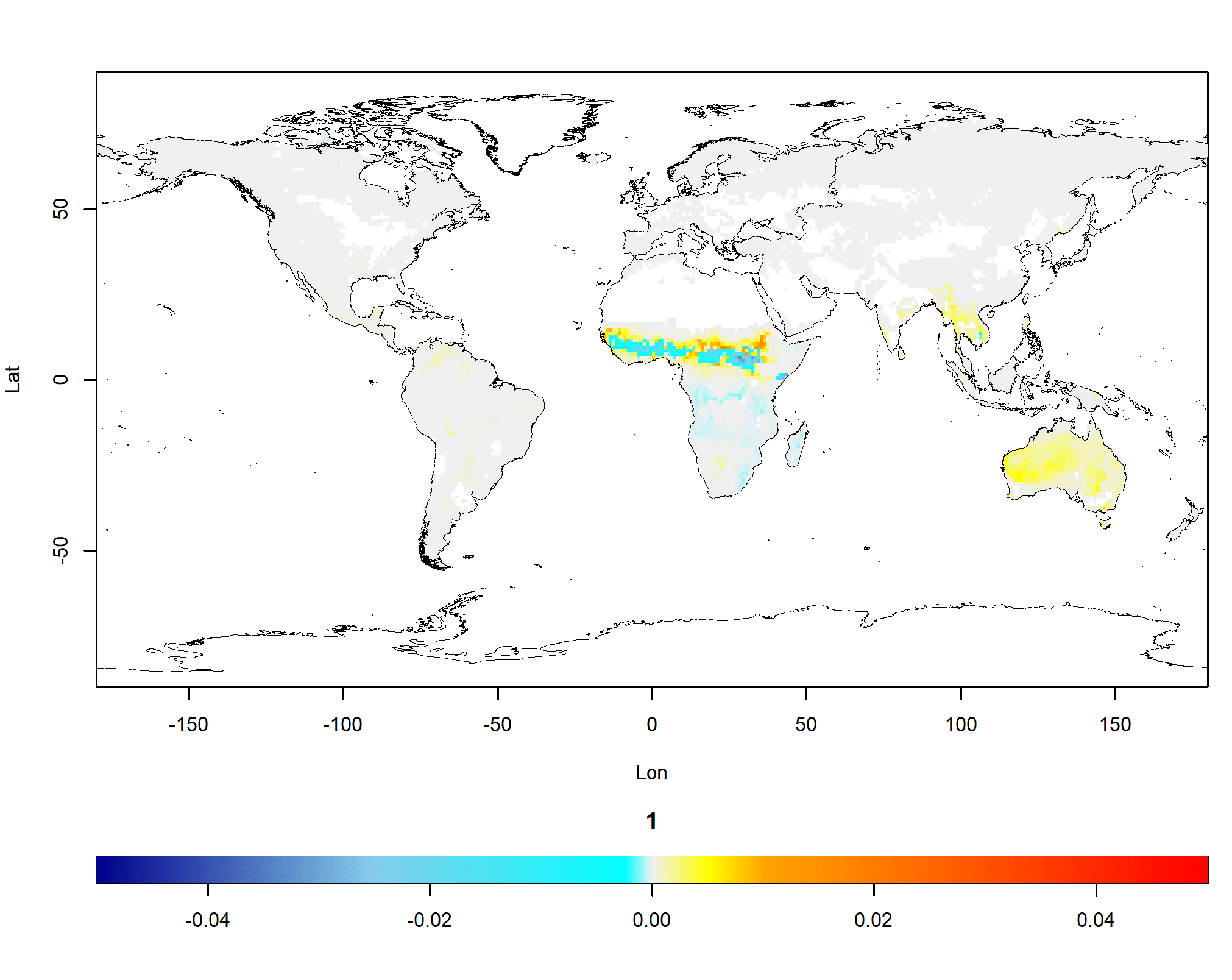

Supplement: Supplementary file 2 — Supplementary material 2 [file 41598_2021_81233_MOESM2_ESM.gif]
